# Ward-level leadership quality and prospective low-back pain of eldercare workers: do resident handlings mediate the association?

**DOI:** 10.1007/s00420-023-01989-2

**Published:** 2023-06-19

**Authors:** Leticia Bergamin Januario, Svend Erik Mathiassen, Andreas Holtermann, Gunnar Bergström, Matthew Leigh Stevens, Reiner Rugulies, David Hallman

**Affiliations:** 1 Department of Occupational Health Science and Psychology, Centre for Musculoskeletal Research, University of Gävle, Kungsbäcksvägen 47, 801 76 Gävle, Sweden; 2 National Research Centre for the Working Environment, Copenhagen, Denmark; 3 Unit of Intervention and Implementation Research for Worker Health, Institute of Environmental Medicine, Karolinska Institutet, Stockholm, Sweden; 4 Department of Public Health, University of Copenhagen, Copenhagen, Denmark; 5 Department of Psychology, University of Copenhagen, Copenhagen, Denmark

**Keywords:** Healthcare, Managers, Patient handling, Psychosocial factors, Musculoskeletal pain, Mediation analysis

## Abstract

**Objective:**

We investigated the extent to which ward-level leadership quality was associated with prospective low-back pain among eldercare workers, and how this association was mediated by observed resident handlings.

**Methods:**

530 Danish eldercare workers, employed in 121 wards, distributed across 20 nursing homes were evaluated. At baseline, leadership quality was measured using the Copenhagen Psychosocial Questionnaire, and resident handlings [handlings per shift, handlings not using assistive devices, handlings done alone, interruptions to handlings, impediments to handlings] were assessed using observations. Frequency and intensity of low-back pain was assessed monthly during the following year. All variables were averaged for each ward. We used ordinary least squares regressions to examine direct effects of leadership on low-back pain and indirect effects through handlings, using PROCESS-macro for SPSS.

**Results:**

After adjustments for low-back pain at baseline, type of ward, staff ratio (i.e., number of workers divided by number of residents) and proportion of devices not in place, leadership quality showed no effect on prospective low-back pain frequency (*β* = 0.01 [− 0.05:0.07]) and a small beneficial effect on pain intensity (*β* = − 0.02 [− 0.04:0.00]). Resident handlings did not mediate the association between leadership quality and frequency or intensity of low-back pain.

**Conclusions:**

Good leadership quality was associated with a small decrease in prospective low-back pain intensity, but resident handlings did not seem to play a mediating role, although better ward-level leadership quality contributed to fewer workplace-observed resident handlings without assistance. Potentially, organizational factors, such as type of ward and staff ratio, may have a greater influence on handlings and low-back pain than leadership quality per se among eldercare workers.

**Supplementary Information:**

The online version contains supplementary material available at 10.1007/s00420-023-01989-2.

## Background

In healthcare occupations, low-back pain is a common and impairing symptom (Fjell et al. 2007; Montano 2016; Wu et al. 2020; Oakman et al. 2020). Low psychosocial resources in the workplace such as low levels of leadership quality, have been associated with increased low-back pain and other health related problems, such as long-term sickness absence (Oakman et al. 2020; Mehta et al. 2022). In eldercare nursing homes specifically, workers perceive that their health is partly dependent on the attributes of their closest managers, including what managers do in the workplace, and how they do it (Vidman and Strömberg 2021). Evidence also suggests that if the workers perceive a good leadership quality, they also report reduced physical demands (Januario et al. 2020). Thus, a good leadership quality can promote good work conditions and even improve workers’ health (Schaufeli 2015; Bakker and Demerouti 2017).

However, the causal mechanisms by which leadership quality (or other psychosocial factors) is related to workers’ health, including low-back pain, are still uncertain (Niedhammer et al. 2021; Swain et al. 2020). Handling of residents is a particularly demanding task in terms of biomechanical exposure for eldercare workers, and several studies acknowledge resident handlings as an important risk factor for the presence of low-back pain (Koppelaar et al. 2011; Holtermann et al. 2013; Noble and Sweeney 2018; Januario et al. 2021). Thus, it appears likely that in work-units, i.e. wards, with good leadership quality, managers would improve working conditions by, for example: having more assistive devices available, promoting courses on handling techniques so less handlings would be done alone, or distributing handlings more evenly among workers. This would make resident handlings less demanding for workers, and consequently lead to less low-back pain. However, evidence is still limited about the role of leadership quality on the presence of low-back pain, and in particular, the possible mediating role of biomechanical exposures (Armstrong et al. 1993; Devereux et al. 1999), such as resident handlings. This should be further investigated (Oakman et al. 2017), for the purpose of eventually identifying adequate prevention and intervention strategies to reduce low-back pain among eldercare workers.

Most previous studies (Schaufeli 2015; Bakker and Demerouti 2017; Oakman et al. 2020; Mehta et al. 2022) have measured leadership quality at the level of individual workers, taking into account each individual’s opinion regarding his/her managers, without considering that several workers have the same manager. Leadership quality should, however, be considered as a workplace characteristic, *in casu* measured at the ward level, with the opinions of all workers under the supervision of a specific manager being averaged into a single score of his/her leadership quality. Averaging scores at the ward level also reduces reporting bias caused by individual assessments (Mehta et al. 2022). Field studies focusing on resident handlings often rely on self-reported biomechanical exposures, which are prone to bias (Podsakoff et al. 2003) and they only consider one single resident handling characteristic at the time as the main exposure (Koppelaar et al. 2011; Holtermann et al. 2013). As an alternative to self-reported data, observational exposure assessment tools have been developed to assess different exposures in eldercare (Jakobsen et al. 2015; Karstad et al. 2018b). Also, several resident handling conditions should be evaluated together, since they are likely to occur concomitantly (Soria-Oliver et al. 2021), such as when handling a resident alone, without any assistive device, and being interrupted by a coworker who needs urgent help.

Therefore, the aim of this study was to investigate the extent to which eldercare workers’ perception of leadership quality at the ward level is associated with low-back pain frequency and intensity among the workers during a one-year follow-up, and how this association is mediated by observed resident handling conditions. We hypothesize that a better perceived leadership quality at the ward level is associated with less low-back pain (direct effect) among eldercare workers and that this effect is mediated by aspects of resident handlings.

## Methods

### Design and study population

This longitudinal study uses data from the Danish Observational Study of Eldercare work and musculoskeletal disorders (DOSES), described elsewhere (Karstad et al. 2018a). In short, this is a multilevel study, in which 83 nursing homes in the Copenhagen region were invited to participate by email and follow-up phone calls, followed by meetings with the nursing home managers and representatives of the staff. After these meetings, 20 nursing homes agreed to participate. In these homes, all workers meeting the inclusion criteria (below) at all wards were invited to participate through informal meetings and a short screening questionnaire including the informed consent form. Data were collected on 530 eldercare workers (59% of all eligible workers), who were employed in 121 wards across the 20 participating nursing homes.

In this cohort, we included eldercare workers between 18 and 65 years of age who were employed more than 15 h per week, worked on days, evenings, or changing shifts, spent at least one-quarter of their working time on tasks related to direct care of residents, and were employed at facilities where the elderly were living (i.e., nursing homes / eldercare homes). Exclusion criteria for participation were long-term sickness absence, pregnancy and not being permanently employed. Baseline data collection included questionnaires and onsite observations of resident handlings, conducted during about 1.5 years when logistically possible, based on the nursing homes’ schedules. After that the observation period, the data on self-reported musculoskeletal pain were collected monthly during an one-year follow-up.

### Measurements

#### Descriptive information

At baseline, both eldercare workers and ward managers received a web-based questionnaire. Ward managers were, in general the closest managers to the eldercare workers, and they were responsible for taking decisions at their own ward, managing the staff and communicating with higher level management. We asked the managers about the type of ward (somatic, dementia, rehabilitation, or psychiatric unit), the staff ratio (number of eldercare workers divided by number of residents) and whether assistive devices were not in place (‘often’ [daily, 1–3 times/week], or ‘rarely’ [2 times/month or 1 time/month or less]). The questionnaire to the eldercare workers asked about age and smoking habits (smoker or nonsmoker), in addition to questions not used in the present study. We calculated body mass index (BMI—kg/m^2^) based on the measured height and weight, collected at a health check session performed at baseline by trained clinical personnel.

#### Leadership quality

At baseline, the eldercare workers answered four questions about leadership quality from the second version of Copenhagen Psychosocial Questionnaire, COPSOQ II (Pejtersen et al. 2010): “To what extent would you say that your immediate superior … (1) makes sure that the individual member of staff has good development opportunities?, (2) gives high priority to job satisfaction?, (3) is good at work planning?, (4) is good at solving conflicts?”. All questions were answered using a 5-point Likert scale (ranging from “5—To a very large extent” to “1—To a very small extent”). The responses were averaged across items and converted to a score of 0–100, whereby higher scores express better quality of leadership. The internal consistency of this scale was considered adequate in our population (Cronbach’s *α* = 0.86) (Adamson and Prion 2013). We calculated the ward level leadership quality as the aggregated mean value of individual scores of leadership within each ward, and assigned that value to all workers within the ward. This aggregation was supported by a considerable (32.5%) variance in average leadership scores between wards. For interpretation purposes, we also stratified the wards into two groups, i.e. leadership levels ‘below the Danish national average’ and ‘above the Danish national average’, on the basis of the Danish national average of 55.3 (Pejtersen et al. 2010).

#### Resident handling conditions

Resident handlings were evaluated during baseline using a reliable observation instrument (Karstad et al. 2018b). Based on the observed manual handling activities and barriers to resident handlings, we identified 5 handling conditions, as described in detail elsewhere (Januario et al. 2021*): number of handlings per shift, number of handlings done without any assistive device, handlings done alone, impediments* and *interruptions*. In brief, assistive devices, such as transfer belts, sliding sheets, drawing sheets, electric turning sheets, floor hoists and ceiling hoists were available at the observed nursing homes, and if the eldercare worker did not use any of these devices, the handling was noted as a *handling done without any assistive device*. If the worker did not get assistance by another person (typically a co-worker or a visitor), it was noted as a *handling done alone*. An interruption was defined as an event that significantly interrupted the eldercare worker in performing a task (e.g., an urgent request for assistance from a co-worker); and an impediment was defined as an obstacle for completing a task that required some effort (e.g., a broken or missing assistive device).

In total, 4716 interactions between the eldercare workers and residents were observed, which most often involved resident handlings. A previous study (Jakobsen et al. 2015) showed that approximately 71% of all resident handling activities occurred during a period of 4 h in the morning and 4–5 h in the evening, and to save resources, we limited the observations in each one of the 126 wards to these two time periods. For the present study, handlings were averaged at each specific ward as a measure of handling conditions at that ward. If no handlings were observed neither in the morning nor in the evening during the day of observation at a given ward, that ward was excluded from our sample. The reason for having no observed handlings in a ward were that residents did not require this type of care or refused to be observed (7% of residents) or that the worker doing the observed handlings refused to participate in the other measurements.

#### Low-back pain

At baseline and monthly over the one-year follow-up, eldercare workers were asked about the frequency of low-back pain experienced in the previous month (“In the last four weeks, how many days did you have pain in your low-back region?”) with possible answers ranging from 0 to 28 days. If the workers answered that they had pain, they were requested to answer about the intensity of that pain (“On a scale from 0 to 10, what was the worst pain you experienced in your low-back within the past four weeks?”), with answers ranging from 0 (“No pain”) to 10 (“Worst possible pain”) (Karstad et al. 2018a), based on the Standardised Nordic questionnaire for musculoskeletal symptoms (Kuorinka et al. 1987). At baseline these questions were answered through a web-based questionnaire, and during follow-up, the workers received the questions in a text message (Johansen and Wedderkopp 2010) once every 28 days throughout a year. In total, the workers answered pain-related questions 14 times. Both frequency and intensity of low-back pain at baseline were aggregated at the ward level by averaging the scores of all the workers in a given ward into one single value. For the follow-up measurements, we calculated the individual average of ratings at all 14 time-points over the year into two single variables, measuring mean frequency and intensity of low-back pain, respectively, per month. Then we averaged this value per ward to obtain the low-back pain values at the ward level. We included wards in the analysis where at least one worker had answered the questions related to low-back pain both at baseline and at least at 3 time-points during follow-up.

### Statistical analysis

**Fig. 1:**
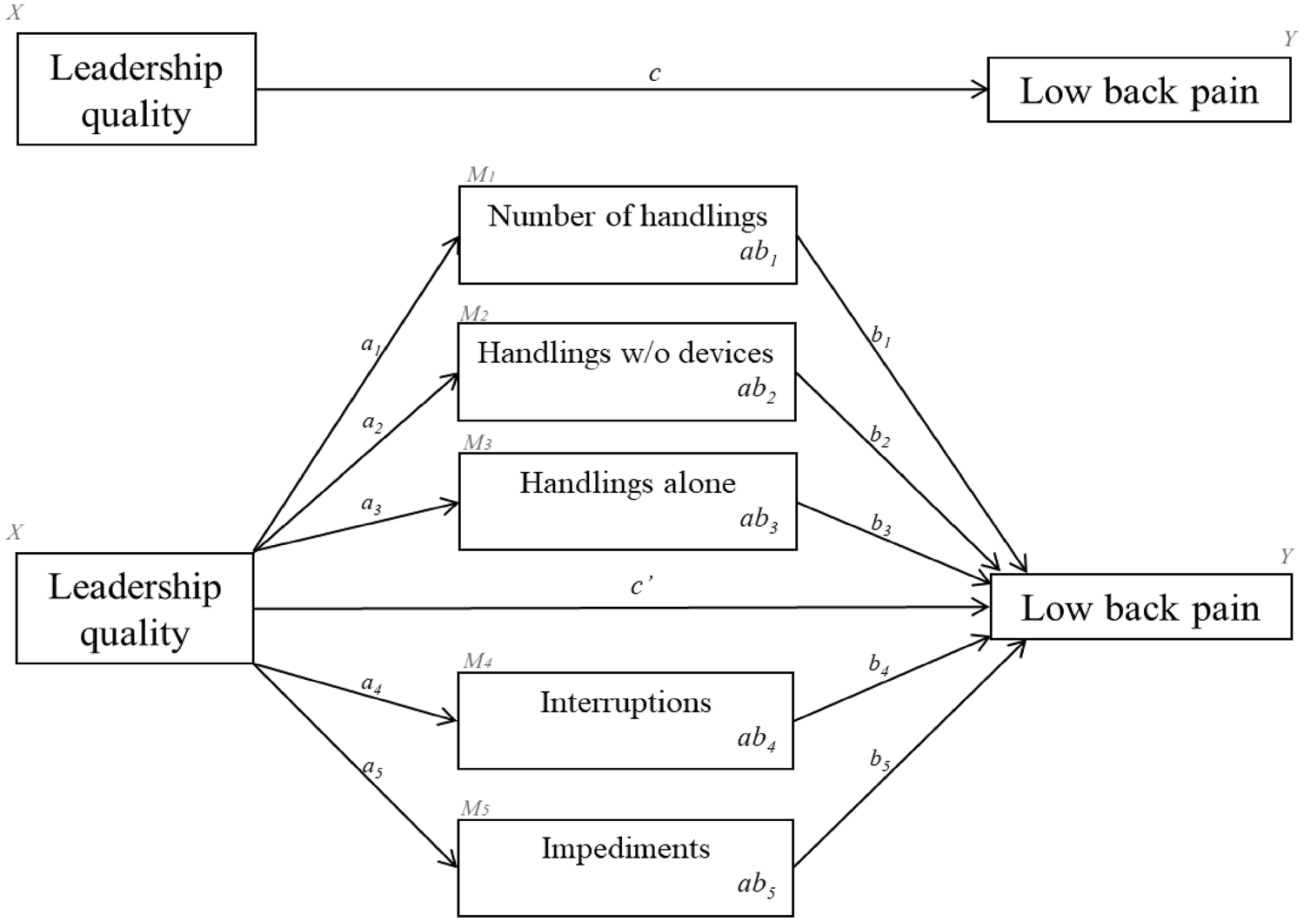
The mediation model showing the total effect (path **c**) of leadership quality (predictor—X) on low-back pain (outcome—Y). In the expanded model (below), path c’ shows the direct effect of leadership quality (X) on low-back pain (Y), which may be mediated through 5 parallel handling conditions: number of handlings per shift (mediator 1—M_1_), handlings without assistive devices (mediator 2—M_2_), handlings done alone (mediator 3—M_3_), interruptions to handlings (mediator 4—M_4_) and impediments to handlings (mediator 5—M_5_), which are represented in each corresponding box by the paths *ab*_*j*_. In the model, *a*_*j*_ show the single effects of leadership quality (X) on each mediator (M_j_) and *b*_*j*_ represent the single effect of each mediator (M_j_) on low-back pain (Y). The model was used for analyzing low-back pain frequency and intensity in two separate analyses

To understand the effect of leadership quality on low-back pain at the ward level, we built a mediation model in which leadership was treated as the exposure variable (*X*), resident handling conditions [*number of handlings, handlings without assistive device, handlings done alone, impediments,* and *interruptions*] as multiple simultaneous mediators (*M*_*1*_ to *M*_*5*_), and low-back pain as the outcome (*Y*), Fig. 1. Low-back pain frequency and intensity were analyzed separately, adjusting each model for their baseline ward-level scores. Besides evaluating both the total and the direct effect of leadership quality on low-back pain (paths *c* and *c’,* respectively, Fig. 1), we analyzed the single effects of leadership on handlings (paths *a*_*1*_ to *a*_*5*_) and the single effects of handlings on low-back pain (paths *b*_*1*_ to *b*_*5*_) using ordinary least squares regressions. The indirect effect of leadership on low-back pain through handling conditions was analyzed for possible mediation by all handling conditions combined (paths *ab*_*total*_) and for each handling condition separately (paths *ab*_*j*_) (Hayes 2021).

We performed all statistical analyses using IBM SPSS Statistics 27.0 (IBM Inc., Chicago, IL, USA) and set p < 0.1. We used descriptive statistics to characterize the sample in terms of work conditions, leadership quality, and low-back pain. To examine relations between handling conditions and leadership quality, we compared wards with leadership level ‘below the Danish national average’ and wards with leadership level ‘above the Danish national average’, using analysis of variance (ANOVA) for continuous variables and Chi-squared tests for descriptive categorical variables. For the mediation analyses, we analyzed a parallel mediation model using the macro PROCESS, version 4.0 (Hayes 2021). Before using the macro, we tested regression assumptions for low-back pain frequency and intensity, considering that ordinary least squares regressions are used to perform the analyses in the PROCESS macro. We centered leadership quality and all five handling conditions on their mean. Depending on the analyzed outcome, we controlled the model for baseline scores of low-back pain frequency and intensity at the ward level, calculated as the average score across workers employed at each ward. In a subsequent step, we adjusted each model for type of ward, staff ratio and proportion of devices not in place, by including them as covariates. We calculated 95% confidence intervals (CI) of the indirect effects of leadership on low-back pain through handling conditions (paths *ab*) by bootstrapping (5000 samples). We used Cribari − Neto’s heteroscedasticity-consistent standard error (HC4) to protect against bias due to violations of the homoscedasticity assumption (Hayes and Cai 2007).

## Results

The eldercare workers were mostly women (90.3%), with mean age 45.4 (SD 10.9) years, BMI 26.5 (SD 5.3) kg/m^2^, and 30% of the population were smokers. Of the 553 workers included in DOSES, information was incomplete for 23; thus, 530 workers distributed across 121 wards were included in further analyses. This corresponds to an average of 4.4 workers (ranging from 1 to 14 workers) per ward. The average ward level leadership quality on the COPSOQ scale was 59.2 (SD between wards: 12.5) of 100, which was above the Danish national average of 55.3 (Table 1).

**Table 1:** Descriptive characteristics of the 121 wards, both for the total population and stratified into wards below and above the Danish national average for quality of leadership. The *P *value shows the statistical significance of this difference

	Wards in total (*N* = 121)		Wards below the national average (*N* = 44)		Wards above the national average (*N* = 77)		*P* value
	*N* (%)	Mean (SD)	*N* (%)	Mean (SD)	*N* (%)	Mean (SD)	
Workers per ward							
Up to 2 workers	26 (21.5)		11 (25.0)		15 (19.5)		0.22
3 to 5 workers	59 (48.8)		23 (52.3)		36 (46.7)		
6 or more workers	36 (29.7)		10 (22.7)		26 (33.8)		
Quality of leadership (0–100)		59.2 (12.5)		46.2 (6.7)		66.7 (8.2)	< 0.01*
Handling conditions per shift							
Number of handlings		29.7 (16.5)		32.3 (17.2)		28.6 (16.0)	0.23
Handlings without devices		16.1 (10.7)		17.3 (11.0)		15.5 (10.5)	0.37
Handlings alone		16.4 (11.4)		18.9 (11.8)		15.1 (11.1)	0.08
Interruptions		10.9 (7.6)		11.4 (7.6)		10.6 (7.8)	0.57
Impediments		8.0 (4.5)		8.1 (4.3)		7.9 (4.6)	0.79
Low-back pain at baseline							
Frequency (0–28 days)		7.6 (5.3)		8.7 (6.8)		7.0 (4.2)	0.08
Intensity (0–10)		3.9 (1.7)		4.2 (1.9)		3.7 (1.6)	0.13
Low-back pain during follow-up^1^							
Frequency (0–28 days)		7.2 (4.9)		8.0 (5.9)		6.7 (4.1)	0.17
Intensity (0–10)		3.5 (1.6)		3.9 (2.0)		3.2 (1.4)	0.03*
Type of ward							0.75
Somatic	91(74.6)		34 (77.3)		57 (74.0)		
Dementia	26 (21.3)		9 (20.5)		16 (20.8)		
Rehabilitation	3 (2.5)		1 (2.3)		2 (2.6)		
Psychiatric unit	2 (1.6)		0 (0.0)		2 (2.6)		
Staff ratio		0.50 (0.30)		0.52 (0.43)		0.47 (0.11)	0.41
Devices not in place							0.05
Often	12 (13.8)		5 (17.9)		7 (11.9)		
Rarely	75 (86.2)		23 (82.1)		52 (88.1)		
Missing values	34 (28.1)		16 (36.4)		18 (23.4)		

*N:* number of wards. *SD:* standard deviation

^1^average values across the follow-up period, including measurements at all 14 time points during the follow-up year

*Statistically significant at *p* < 0.05

For wards having a ‘below the national average’ leadership quality, handling conditions were worse compared to wards with ‘above the national average’ leadership, with a borderline significant difference for the number of handlings done alone, and ward managers reported that assistive devices were more often not in place. In wards with ‘below the national average’ leadership quality, the workers also reported more days with low-back pain at baseline and higher low-back pain intensity during follow-up.

**Fig. 2:**
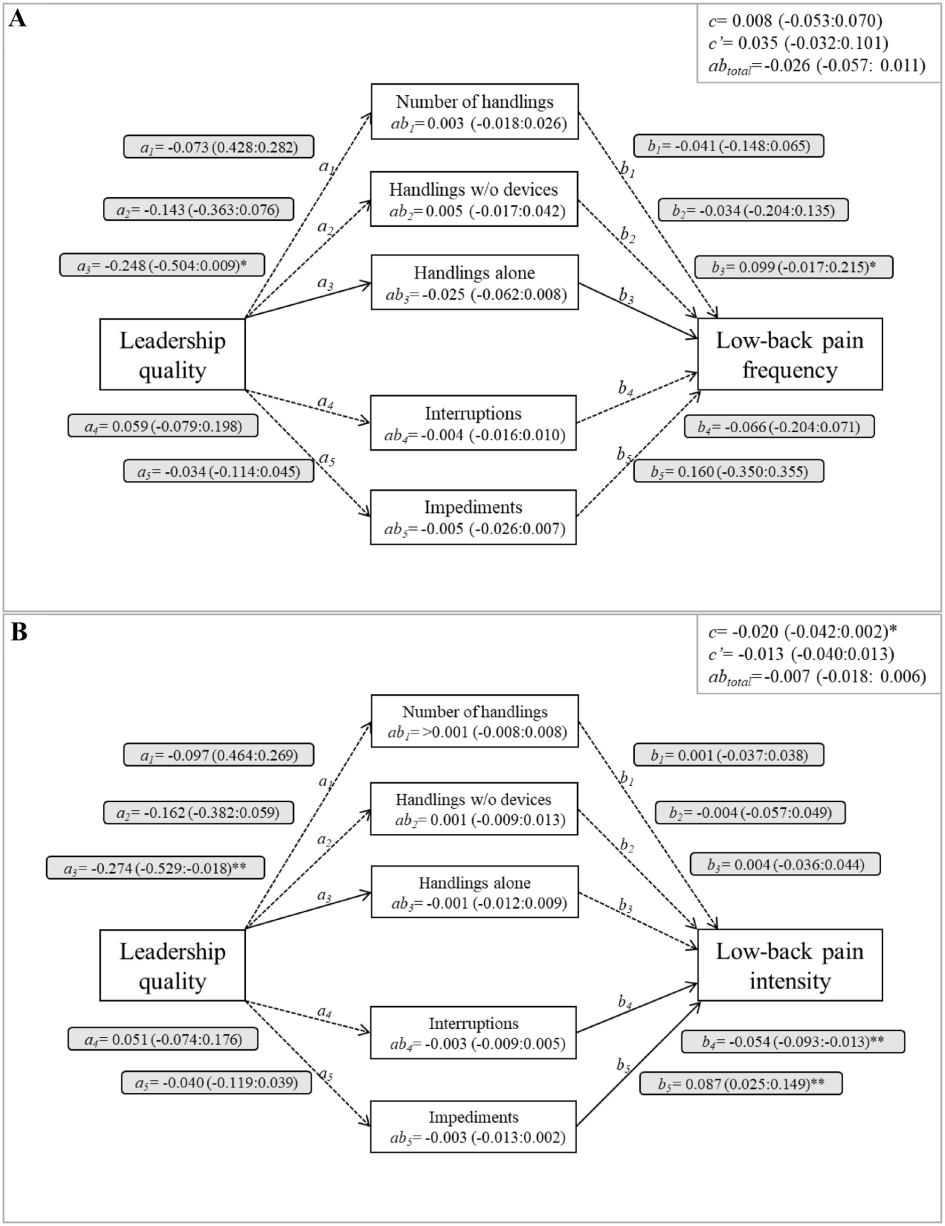
Estimated effects (with 95% confidence intervals) for the mediation analysis of (**A**) frequency and (**B**) intensity of low-back pain over one year, adjusted for baseline values of pain, type of ward, staff ratio and proportion of devices not in place. The boxes in the upper right corner show the main results of the mediation analysis, including the total effect (path *c* in Fig. 1), the indirect effect (path *c’* in Fig. 1) and the total indirect effect (path *ab*_*total*_). Each individual indirect effect (path *ab*_*j*_) is shown in the box corresponding to each handling condition. Single regression results are shown in grey boxes, measured cross-sectionally in path *a*_*j*_ and longitudinally in path *b*_*j.*_ *p < 0.1, **p < 0.05

The mediation analysis (Fig. 2A) showed that leadership quality at the ward level was not significantly associated with average low-back pain frequency during the subsequent year (total effect –path c). None of the observed handling conditions mediated the association, since for both total indirect effect (path *ab*_*total*_) and all individual indirect effects (paths *ab*_*j*_), confidence intervals on β included zero, both in the unadjusted and in the adjusted models (detailed information in supplementary table S1). Single regressions (Fig. 2A, grey boxes) showed that, after adjustments, higher quality of leadership was, in general, associated with more favorable handling conditions (path *a*), which in turn were associated with lower frequency of low-back pain (path *b*). Specifically, high quality of leadership was associated with less *handlings done alone* (path *a*_*3*_, *p* = 0.05, measured cross-sectionally) and high number of *handlings done alone* was associated with higher low-back pain frequency (path *b*_*3*_, *p* = 0.08, measured longitudinally). For the unadjusted single regressions, high number of *impediments* was associated with higher low-back pain frequency (path *b*_*5*_: *β* = 0.149; CI − 0.019 to 0.311; *p* = 0.08, measured longitudinally; supplementary table S1).

For low-back pain intensity (Fig. 2B), leadership quality had a borderline negative total effect (path *c*) in the adjusted model, indicating that good leadership lead to less low-back pain intensity, but no statistically significant direct effect was found (path c’) and both the total indirect effect (path *ab*_*total*_) and all individual indirect effects (paths *ab*_*j*_) included zeros in their confidence intervals, indicating that handling conditions did not mediate the association between leadership quality and low-back pain intensity. Single regressions (Fig. 2B, grey boxes) showed that a good leadership quality was associated with less *handlings done alone* (path *a*_*3*_, p = 0.03, measured cross-sectionally) and that a high number of interruptions (path *b*_*4*_, p = 0.01) and a high number of impediments (path *b*_*5*_, p = 0.01) were associated with more low-back pain intensity during the one-year follow-up. For the unadjusted single regressions, high number of *impediments* was associated with higher low-back pain intensity (path *b*_*5*_: *β* = 0.061; CI: − 0.001 to 0.121; *p* = 0.04, measured longitudinally; supplementary table S2).

## Discussion

Our study aimed to understand the role of leadership quality, an important psychosocial factor, for the occurrence of low-back pain at the ward level during a one-year follow-up, and to consider resident handlings, which are biomechanically demanding and common among eldercare workers, as potential mediators. On the contrary to our initial hypothesis, a good leadership quality was associated with only a small decrease in low-back pain intensity during the one-year follow-up and resident handlings did not seem to play a mediating role in this association, although better ward-level leadership quality contributed to fewer workplace-observed resident handlings without assistance.

### Leadership quality and resident handlings

Leadership quality had little or no effect on handling conditions. Based on theoretical models on leadership quality (Schaufeli 2015; Bakker and Demerouti 2017), we expected that in wards with higher perceived leadership quality, we would also find better working conditions, as expressed in better resident handling conditions. However, among the five investigated handling conditions, only *handlings done alone* was associated with leadership quality, after adjustments for baseline values of pain, type of ward, staff ratio and proportion of devices not in place (path *a*_*3*_ in Fig. 2). Increasing the ward level leadership quality by 5 points (on the 0–100 scale) would decrease the number of handlings alone per shift by approximately 1.3. A previous study showed that healthcare workers with high risk of developing persistent low-back pain due to a high frequency of resident handlings per shift, reported lower leadership quality than those without a risk (Holtermann et al. 2013). To our knowledge, no other study has considered the effects that leadership quality may have on whether resident handlings are performed alone or with the assistance of co-workers.

### Resident handlings and low-back pain

The association between resident handling conditions and the prevalence of low-back pain is well established in the literature (Koppelaar et al. 2011; Holtermann et al. 2013; Noble and Sweeney 2018; Januario et al. 2021). However, in our study we found only small associations with low-back pain of *handlings done alone*, *impediments* and *interruptions* in the single regression analyses after adjustments. For example, having 10 more *handlings alone* in a shift would lead to approximately one day increase in low-back pain frequency per month at follow-up, and having 5 more *impediments* in a shift would lead to an increase of approximately 0.5 units in low-back pain intensity per month. Considering that the follow-up is only one year, larger effects may be expected at a longer term. In addition, it is important to realize that the present study operated at the ward level in order to understand the effects of factors in the work environment that are shared by the workers. Although the ward-level approach highlights these shared factors, it also levels out possible individual ‘extremes’, e.g. in the number of handlings, that may have influenced the associations found in previous studies performed at the individual level. The data on exposures were also based on quite few workers in many wards (70.3% of the wards had 5 or less participating workers), and the ward-level mean values are thus subject to uncertainty. This may have contributed to the small and nonsignificant associations with low back pain found in the present study.

The DOSES observational instrument considers broken or missing assistive devices as an impediment, and an urgent request for assistance from a coworker as an interruption, and both can be considered as barriers that hamper or disrupt the completion of a task, and thus have a negative impact on health (Griffin et al. 2007; Rau et al. 2010). Resumption of a task after the removal of a barrier requires increased work pace, which can lead to errors and increased workload to complete the task during the same time slot as if it had not been interrupted or impeded. In our study, barriers occurred at approximately 65% of all handlings during a shift (Table 1: approximately 30 handlings per shift, with 11 interruptions and 8 impediments). This may lead to stress and maybe musculoskeletal pain (Flynn et al. 1999; Mark et al. 2008). However, in our study, even though only to small extent, interruptions were associated with decreased low-back pain intensity (an increase by 5 interruptions per shift would lead to a decrease in 0.5 units in low-back pain intensity per month at follow-up). It is possible that more interruptions is a proxy for workers more often asking colleagues for assistance, which could, in turn, result in fewer handlings performed alone and/or the use of adequate assistive devices, which would be protective against low-back pain (Nelson et al. 2006; Kanaskie and Snyder 2018). A previous study of eldercare workers showed that barriers were not associated with health problems (Jakobsen et al. 2015) and that asking colleagues for assistance could increase the perception of social support and collaboration at work (Jakobsen et al. 2018).

### Leadership quality and low-back pain, with resident handlings as mediators

Leadership quality was associated with less overall mean low-back pain intensity at the ward level at follow-up (Fig. 2B, C), but only to a very small extent: an increase of 10 units in perceived ward-level leadership quality (on the 0–100 scale) leads to a decrease in pain intensity of 0.2 units per month (on the 0–10 scale). This decrease is way smaller than the 2.0 units proposed by (Ostelo and de Vet 2005) as the minimal important clinical difference. However, this result should still be acknowledged, considering that larger effects may be expected at a longer term (i.e., periods longer than a one-year follow-up).On the contrary to our hypothesis, none of the resident handling conditions mediated the association between leadership and low-back pain, irrespective of whether its frequency or intensity was assessed. Other studies have shown that low leadership quality is associated with multisite musculoskeletal pain, if not specifically in the low back (Fjell et al. 2007; Oakman et al. 2020), but to our knowledge no other study has considered whether resident handlings could mediate this association.

A previous study evaluated the effects of psychosocial factors, including leadership, on musculoskeletal symptoms, mediated through psychological strain (i.e. anger, anxiety, depression, and frustration) and found a mediation effect of strain in the association between leadership and wrist/hands symptoms, while no associations were found for low-back symptoms (Eatough et al. 2012). The authors suggested that other causal mechanisms than psychological strain may explain that leadership and musculoskeletal symptoms are associated, and that the physical work environment might be a candidate (Eatough et al. 2012). In our study, we did investigate biomechanical exposures, i.e., resident handlings, but we could not verify any effect on low-back pain. In addition, we considered leadership quality as an indicator of a job resource that is shared between eldercare workers in a ward. Drawing on the job demands − resources model (Bakker and Demerouti 2017) and previous research on psychosocial (Niedhammer et al 2021) and biomechanical risk factors for low back pain (Swain et al. 2020), we expected that better leadership quality would contribute to reduced symptoms of low back pain in eldercare wards and that this would be partly mediated by resident handlings, which reflect relevant biomechanical exposures (Swain et al. 2020). However, our longitudinal ward-level data on more than 500 workers did not support this hypothesis. Thus, clarifying the extent to which psychosocial and physical exposures act together in determining the occurrence of musculoskeletal symptoms is a challenge that requires further research.

By assessing leadership quality through COPSOQ, we evaluated the workers’ perception of their managers in terms of preferable leadership, considering factors, such as development opportunities, job satisfaction, work planning and ability to solve conflicts (Pejtersen et al. 2010). Even though COPSOQ is a validated and widely used method to evaluate psychosocial factors, it is important to consider that leadership quality is a broad characteristic of the work environment and that other aspects of leadership beyond those evaluated in COPSOQ may impact resident handling conditions and pain, for example leadership styles (change-oriented style, production/structured-oriented style, employee relations-oriented style) (Fjell et al. 2007), or safety-specific leadership, i.e. that managers set safety-specific goals for the workers, reward safety-related behaviors, and coach workers to perform job tasks safety (Eatough et al. 2012). A previous study of the same DOSES cohort used in the present study concluded that the leaders’ knowledge and behaviors towards workers’ pain were associated with future musculoskeletal pain and sickness absence, but also that it is important to consider biopsychosocial aspects, such as communication between manager and employment, which may influence musculoskeletal symptoms (Rasmussen et al. 2022).

### Methodological considerations

Strengths of this study include the use of observational tools to evaluate resident handlings, assessment of pain longitudinally, and addressing the characteristics of the ward, not the specific individual, in the analyses. By aggregating all exposures at the ward level, we overcame individual worker variability and a potential risk of bias. Even considering pain as a ward level measure is relevant, because symptomatic workers may have an impact on the distribution of work and consequently the workload for the whole group in a particular ward. The evaluation of work conditions at a supra-individual level, such as a group, ward or organization, is still sparse in the literature when assessing psychosocial factors and their potential effects on, e.g. productivity and well-being (Roczniewska et al. 2021) and our study contributes to understanding the effects of supra-individual quality of leadership on musculoskeletal pain.

However, some limitations should also be acknowledged. We did not discriminate between types of shifts (day or evening) in our analysis, even though both resident handlings and leadership may be different in different shifts. We also emphasize that the analysis was focused on ward-level exposures and did not specifically consider individual factors that may be associated with low-back pain, including years of employment, additional or previous jobs, and lifestyle factors, such as leisure-time physical activity. Differences between wards in these factors and the associated effects on low-back pain over time will appear as larger errors in the statistical models. We did, however, adjust our models for low-back pain at baseline, which may represent the integrated effect of the mentioned individual factors.

Although we found only little direct effect of leadership quality on low-back pain, and no indirect effects through resident handlings, we believe that the lack of statistically significant results is not a consequence of an underpowered study, considering that we included 121 wards in our sample. Using data on the ward level reduces the effect of individual ‘outliers’, but we may have maintained some instability in our results, considering that some of the wards contributed to the study with very few workers.

In addition, it is important to consider that other factors, such as work distribution, time pressure and lack of knowledge on how to handle technology-based assistive devices may have a stronger impact on low-back pain through handling conditions (Lee et al. 2010; Noble and Sweeney 2018) than leadership quality per se, and future studies should investigate this further.

## Conclusions

Our study showed that good perceived leadership quality was associated with lower low-back pain intensity during a one-year follow-up, but only to a very small extent after adjustment for several organizational factors. Resident handlings did not play a mediating role in this association. These results led us to believe that other factors than leadership quality, such as the work organization in terms of the distribution of tasks at the ward, the staff ratio or even the type of ward in which the eldercare workers are allocated, may have a greater impact on low-back pain through resident handling conditions. This needs to be assessed in future research.

## Supplementary Information

Below is the link to the electronic supplementary material.

Supplementary file1 (DOCX 23 kb)

## Data Availability

The datasets generated during and/or analyzed during the current study are not publicly available in order to protect the anonymity of the participants and ensure that the GDPR is being followed, therefore data sharing is not available in the current format. Please contact the corresponding author for further information.

## References

[CR1] Adamson KA, Prion S (2013). Reliability: measuring internal consistency using cronbach’s α. Clin Simul Nurs.

[CR2] Armstrong TJ, Buckle P, Fine JF (1993). A conceptual model for work-related neck and upper-limb musculoskeletal disorders. Scand J Work Environ Heal.

[CR3] Bakker AB, Demerouti E (2017). Job demands−resources theory: taking stock and looking forward. J Occup Health Psychol.

[CR4] Devereux JJ, Buckle PW, Vlachonikolis IG (1999). Interactions between physical and psychosocial risk factors at work increase the risk of back disorders: an epidemiological approach. Occup Environ Med.

[CR5] Eatough EM, Way JD, Chang CH (2012). Understanding the link between psychosocial work stressors and work-related musculoskeletal complaints. Appl Ergon.

[CR6] Fjell Y, Österberg M, Alexanderson K (2007). Appraised leadership styles, psychosocial work factors, and musculoskeletal pain among public employees. Int Arch Occup Environ Health.

[CR7] Flynn EA, Barker KN, Gibson JT (1999). Impact of interruptions and distractions on dispensing errors in an ambulatory care pharmacy. Am J Heal Pharm.

[CR8] Griffin JM, Greiner BA, Stansfeld SA, Marmot M (2007). The effect of self-reported and observed job conditions on depression and anxiety symptoms: a comparison of theoretical models. J Occup Health Psychol.

[CR9] Hayes AF, Cai L (2007). Using heteroskedasticity-consistent standard error estimators in OLS regression: an introduction and software implementation. Behav Res Methods.

[CR10] Hayes AF (2021) The PROCESS macro for SPSS, SAS, and R. Andrew F. Hayes. Accessed 5 Oct 2021

[CR11] Holtermann A, Clausen T, Jørgensen MB (2013). Patient handling and risk for developing persistent low-back pain among female healthcare workers. Scand J Work Environ Health.

[CR12] Jakobsen LM, Jorgensen AFB, Thomsen BL (2015). A multilevel study on the association of observer-assessed working conditions with depressive symptoms among female eldercare workers from 56 work units in 10 care homes in Denmark. BMJ Open.

[CR13] Jakobsen LM, Albertsen K, Jorgensen AFB (2018). Collaboration among eldercare workers: barriers, facilitators and supporting processes. Scand J Caring Sci.

[CR14] Januario LB, Stevens ML, Mathiassen SE (2020). Combined effects of physical behavior compositions and psychosocial resources on perceived exertion among eldercare workers. Ann Work Expo Heal.

[CR15] Januario LB, Mathiassen SE, Stevens ML (2021). Are resident handlings in eldercare wards associated with musculoskeletal pain and sickness absence among the workers? A prospective study based on onsite observations. Scand J Work Environ Health.

[CR16] Johansen B, Wedderkopp N (2010). Comparison between data obtained through real-time data capture by SMS and a retrospective telephone interview. Chiropr Osteopat.

[CR17] Kanaskie ML, Snyder C (2018). Nurses and nursing assistants decision-making regarding use of safe patient handling and mobility technology : A qualitative study. Appl Nurs Res.

[CR18] Karstad K, Jørgensen AFB, Greiner BA (2018). Danish Observational Study of Eldercare work and musculoskeletal disorderS (DOSES): a prospective study at 20 nursing homes in Denmark. BMJ Open.

[CR19] Karstad K, Rugulies R, Skotte J (2018). Inter-rater reliability of direct observations of the physical and psychosocial working conditions in eldercare: An evaluation in the DOSES project. Appl Ergon.

[CR20] Koppelaar E, Knibbe JJ, Miedema HS, Burdorf A (2011). Individual and organisational determinants of use of ergonomic devices in healthcare. Occup Environ Med.

[CR21] Kuorinka I, Jonsson B, Kilbom a, (1987). Standardised Nordic questionnaires for the analysis of musculoskeletal symptoms. Appl Ergon.

[CR22] Lee SJ, Faucett J, Gillen M (2010). Factors associated with safe patient handling behaviors among critical care nurses. Am J Ind Med.

[CR23] Mark G, Gudith D, Klocke U (2008). The cost of interrupted work: more speed and stress. Conf Hum Factors Comput Syst - Proc Doi.

[CR24] Mehta AJ, Mathisen J, Nguyen T-L (2022). Chronic disorders, work-unit leadership quality and long-term sickness absence among 33 025 public hospital employees. Scand J Work Environ Health.

[CR25] Montano D (2016). Supervisor behaviour and its associations with employees’ health in Europe. Int Arch Occup Environ Health.

[CR26] Nelson A, Matz M, Chen F (2006). Development and Evaluation of a Multifaceted Ergonomics Program to Prevent Injuries Associated with Patient Handling Tasks..

[CR27] Niedhammer I, Bertrais S, Witt K (2021). Psychosocial work exposures and health outcomes: a meta-review of 72 literature reviews with meta-analysis. Scand J Work Environ Health.

[CR28] Noble NL, Sweeney NL (2018). Barriers to the Use of Assistive Devices in Patient Handling. Workplace Health Saf.

[CR29] Oakman J, de Wind A, van den Heuvel SG, van der Beek AJ (2017). Work characteristics predict the development of multi-site musculoskeletal pain. Int Arch Occup Environ Health.

[CR30] Oakman J, Stevens M, Karstad K (2020). Do organisational and ward-level factors explain the variance in multi-site musculoskeletal pain in eldercare workers? A multi-level cross-sectional study. Int Arch Occup Environ Health.

[CR31] Ostelo RWJG, de Vet HCW (2005). Clinically important outcomes in low back pain. Best Pract Res Clin Rheumatol.

[CR32] Pejtersen JH, Kristensen TS, Borg V, Bjorner JB (2010). The second version of the Copenhagen Psychosocial Questionnaire. Scand J Public Health.

[CR33] Podsakoff PM, MacKenzie SB, Lee J-Y, Podsakoff NP (2003). Common method biases in behavioral research: a critical review of the literature and recommended remedies. J Appl Psychol.

[CR34] Rasmussen CDN, Oakman J, Karstad K (2022). Pain management in eldercare employees – the role of managers in addressing musculoskeletal pain and pain-related sickness absence. BMC Public Health.

[CR35] Rau R, Morling K, Rösler U (2010). Is there a relationship between major depression and both objectively assessed and perceived demands and control?. Work Stress.

[CR36] Roczniewska M, Smoktunowicz E, Calcagni CC (2021). Beyond the Individual: A Systematic Review of the Effects of Unit-Level Demands and Resources on Employee Productivity, Health, and Well-Being. J Occup Health Psychol.

[CR37] Schaufeli WB (2015). Engaging leadership in the job demands−resources model. Career Dev Int.

[CR38] Soria-Oliver M, López JS, Torrano F, García-González G (2021). Do psychosocial factors mediate the appearance of musculoskeletal symptoms? Evidence of an empirical study about the role of mental workload in computer workers. PLoS ONE.

[CR39] Swain CTV, Pan F, Owen PJ (2020). No consensus on causality of spine postures or physical exposure and low back pain: A systematic review of systematic reviews. J Biomech.

[CR40] Vidman Å, Strömberg A (2021). Leadership for a healthy work environment—a question about who, what and how. Leadersh Heal Serv.

[CR41] Wu A, March L, Zheng X (2020). Global low back pain prevalence and years lived with disability from 1990 to 2017: estimates from the Global Burden of Disease Study 2017. Ann Transl Med.

