# Supplementary material for: Ward-level leadership quality and prospective low-back pain of eldercare workers: do resident handlings mediate the association?
Source: Int Arch Occup Environ Health. 2023 Jun 19;96(7):1049–59. doi: 10.1007/s00420-023-01989-2 (PMC10361909; doi:10.1007/s00420-023-01989-2)
Supplement: Supplementary file 1 — Supplementary file1 (DOCX 23 kb) [file 420_2023_1989_MOESM1_ESM.docx]

**Supplementary File**

| Table S1. Total, direct, and indirect effects of leadership quality on low-back pain frequency and intensity when including handling conditions as potential mediators. The table even shows results of single regressions considering the effects of leadership quality on handling conditions (path a) and effects of handling conditions on low-back pain (path b). | | | | | | | | | | | |
| --- | --- | --- | --- | --- | --- | --- | --- | --- | --- | --- | --- |
| Path |  | Unadjusted model^1^ | | | | | Adjusted model^2^ | | | | |
|  |  | β | SE | p | LLCI | UUCI | β | SE | p | LLCI | UUCI |
| Total, direct, and indirect effects of leadership on low-back pain frequency (cf. figure 1 and figure 2A) | | | | | | | | | | | |
| *c* | Total effect | 0.001 | 0.027 | 0.98 | -0.053 | 0.055 | 0.008 | 0.031 | 0.78 | -0.053 | 0.070 |
| c' | Direct effect | 0.002 | 0.031 | 0.94 | -0.059 | 0.064 | 0.035 | 0.033 | 0.30 | -0.032 | 0.101 |
| *ab_total_* | Indirect effects | -0.001 | 0.012 | -- | -0.027 | 0.019 | -0.026 | 0.017 | -- | -0.057 | 0.011 |
| *ab_1_* | Number of handlings | <0.001 | 0.006 | -- | -0.016 | 0.011 | 0.003 | 0.010 | -- | -0.018 | 0.026 |
| *ab_2_* | Handlings without devices | 0.003 | 0.008 | -- | -0.012 | 0.023 | 0.005 | 0.014 | -- | -0.017 | 0.042 |
| *ab_3_* | Handlings alone | -0.004 | 0.009 | -- | -0.024 | 0.013 | -0.025 | 0.018 | -- | -0.062 | 0.008 |
| *ab_4_* | Interruptions | 0.002 | 0.005 | -- | -0.007 | 0.013 | -0.004 | 0.006 | -- | -0.016 | 0.010 |
| *ab_5_* | Impediments | -0.003 | 0.006 | -- | -0.018 | 0.006 | -0.005 | 0.008 | -- | -0.026 | 0.007 |
| Effects of leadership on handling conditions (single regressions) | | | | | | | | | | | |
| *a1* | Number of handlings | -0.021 | 0.137 | 0.88 | -0.293 | 0.251 | -0.073 | 0.178 | 0.68 | -0.428 | 0.282 |
| *a2* | Handlings without devices | -0.096 | 0.079 | 0.23 | -0.253 | 0.061 | -0.143 | 0.110 | 0.20 | -0.363 | 0.076 |
| *a3* | Handlings alone | -0.113 | 0.087 | 0.20 | -0.286 | 0.059 | **-0.248** | **0.129** | **0.05** | **-0.504** | **0.009** |
| *a4* | Interruptions | 0.049 | 0.064 | 0.45 | -0.079 | 0.177 | 0.059 | 0.069 | 0.39 | -0.079 | 0.198 |
| *a5* | Impediments | -0.020 | 0.032 | 0.53 | -0.084 | 0.044 | -0.034 | 0.039 | 0.40 | -0.114 | 0.045 |
| Effects of handling conditions on low-back pain frequency (single regressions) | | | | | | | | | | | |
| *b1* | Number of handlings | 0.002 | 0.052 | 0.97 | -0.102 | 0.106 | -0.041 | 0.053 | 0.44 | -0.148 | 0.065 |
| *b2* | Handlings without devices | -0.034 | 0.065 | 0.60 | -0.164 | 0.096 | -0.034 | 0.085 | 0.69 | -0.204 | 0.135 |
| *b3* | Handlings alone | 0.033 | 0.064 | 0.60 | -0.094 | 0.160 | **0.099** | **0.058** | **0.09** | **-0.017** | **0.215** |
| *b4* | Interruptions | 0.041 | 0.063 | 0.51 | -0.083 | 0.166 | -0.066 | 0.069 | 0.34 | -0.204 | 0.071 |
| *b5* | Impediments | **0.146** | **0.083** | **0.08** | **-0.019** | **0.311** | 0.160 | 0.098 | 0.11 | -0.350 | 0.355 |
| 1. Model including baseline values of pain. 2. Model including baseline values of pain and adjusted by type of ward, staff ratio and proportion of devices not in place. | | | | | | | | | | | |

| Table S2. Total, direct, and indirect effects of leadership quality on low-back pain intensity when including handling conditions as potential mediators and single regression considering the effects of leadership quality on handling conditions (path a) and effects of handling conditions on low-back pain (path b). | | | | | | | | | | | |
| --- | --- | --- | --- | --- | --- | --- | --- | --- | --- | --- | --- |
| Path |  | Unadjusted model^1^ | | | | | Adjusted model^2^ | | | | |
|  |  | β | SE | p | LLCI | UUCI | β | SE | p | LLCI | UUCI |
| Total, direct, and indirect effects of leadership on low-back pain intensity (cf. figure 1 and figure 2B) | | | | | | | | | | | |
| *c* | Total effect | -0.017 | 0.011 | 0.11 | -0.038 | 0.004 | **-0.020** | **0.011** | **0.07** | **-0.042** | **0.002** |
| c' | Direct effect | -0.018 | 0.011 | 0.11 | -0.040 | 0.004 | -0.013 | 0.013 | 0.32 | -0.040 | 0.013 |
| *ab_total_* | Indirect effects | -0.001 | 0.012 | -- | -0.027 | 0.019 | -0.007 | 0.006 | -- | -0.018 | 0.006 |
| *ab_1_* | Number of handlings | 0.001 | 0.004 | -- | -0.07 | 0.008 | >0.001 | 0.004 | -- | -0.008 | 0.008 |
| *ab_2_* | Handlings without devices | 0.003 | 0.008 | -- | -0.012 | 0.023 | 0.001 | 0.005 | -- | -0.009 | 0.013 |
| *ab_3_* | Handlings alone | -0.004 | 0.009 | -- | -0.024 | 0.013 | -0.001 | 0.005 | -- | -0.012 | 0.009 |
| *ab_4_* | Interruptions | 0.002 | 0.005 | -- | -0.007 | 0.013 | -0.003 | 0.003 | -- | -0.009 | 0.005 |
| *ab_5_* | Impediments | -0.003 | 0.006 | -- | -0.018 | 0.006 | -0.003 | 0.003 | -- | -0.013 | 0.002 |
| Effects of leadership on handling conditions (single regressions) | | | | | | | | | | | |
| *a1* | Number of handlings | -0.001 | 0.140 | 0.99 | -0.279 | 0.277 | -0.097 | 0.184 | 0.60 | -0.464 | 0.269 |
| *a2* | Handlings without devices | -0.080 | 0.081 | 0.33 | -0.241 | 0.081 | -0.162 | 0.110 | 0.15 | -0.382 | 0.059 |
| *a3* | Handlings alone | -0.097 | 0.090 | 0.28 | -0.276 | 0.082 | **-0.274** | **0.128** | **0.03** | **-0.529** | **-0.018** |
| *a4* | Interruptions | 0.058 | 0.062 | 0.35 | -0.065 | 0.181 | 0.051 | 0.063 | 0.42 | -0.074 | 0.176 |
| *a5* | Impediments | -0.016 | 0.033 | 0.63 | -0.080 | 0.049 | -0.040 | 0.039 | 0.32 | -0.119 | 0.039 |
| Effects of handling conditions on low-back pain intensity (single regressions) | | | | | | | | | | | |
| *b1* | Number of handlings | 0.019 | 0.019 | 0.32 | -0.019 | 0.057 | 0.001 | 0.019 | 0.97 | -0.037 | 0.038 |
| *b2* | Handlings without devices | -0.033 | 0.027 | 0.23 | -0.087 | 0.021 | -0.004 | 0.027 | 0.87 | -0.057 | 0.049 |
| *b3* | Handlings alone | 0.004 | 0.015 | 0.77 | -0.026 | 0.035 | 0.004 | 0.020 | 0.82 | -0.036 | 0.044 |
| *b4* | Interruptions | -0.010 | 0.017 | 0.56 | -0.044 | 0.024 | **-0.054** | **0.020** | **0.01** | **-0.093** | **-0.013** |
| *b5* | Impediments | **0.061** | **0.030** | **0.04** | **0.001** | **0.121** | **0.087** | **0.031** | **0.01** | **0.025** | **0.149** |
| 1. Model including baseline values of pain. 2. Model including baseline values of pain and adjusted by type of ward, staff ratio and proportion of devices not in place. | | | | | | | | | | | |
